# Salivary miRNAs as auxiliary liquid biopsy biomarkers for diagnosis in patients with oropharyngeal squamous cell carcinoma: a systematic review and meta-analysis

**DOI:** 10.3389/fgene.2024.1352838

**Published:** 2024-03-11

**Authors:** Huy Nguyen, Taichiro Nonaka

**Affiliations:** ^1^ School of Medicine, Louisiana State University Health Shreveport, Shreveport, LA, United States; ^2^ Department of Cellular Biology and Anatomy, Louisiana State University Health Sciences Center, Shreveport, LA, United States; ^3^ Feist-Weiller Cancer Center, Louisiana State University Health Shreveport, Shreveport, LA, United States

**Keywords:** head and neck cancer, saliva diagnostics, saliva exosomics, liquid biopsy, circulating biomarker

## Abstract

**Objective:** The healthcare system needs a novel approach to improve and diagnose early oropharyngeal squamous cell carcinoma against its low survival rate. We conduct a systematic review and a comprehensive meta-analysis for the diagnostic role of blood and salivary microRNAs (miRNAs).

**Methods:** An unbiased and thorough literature search in PubMed yielded appropriate data from qualified articles regarding different miRNA biomarkers, method of extraction, research location, and year of publication. Stata was used to calculate the sensitivity, specificity, diagnostic odds ratio, and summary receiver operating characteristic curve.

**Results:** We included 9 studies with 399 qualified oropharyngeal squamous cell carcinoma patients, which yielded a high diagnostic accuracy of blood miRNAs in combination with salivary miRNAs with a sensitivity of 0.70 (*p* < 0.001), specificity of 0.75 (*p* = 0.26), diagnostic odds ratio of 7, and an area under the curve of 0.78.

**Conclusion:** Combined blood- and saliva-derived miRNAs demonstrated a high diagnostic accuracy in detecting oropharyngeal squamous cell carcinoma.

**Systematic review registration:** https://www.crd.york.ac.uk/prospero/display_record.php?ID=CRD42024509424.

## 1 Introduction

With the increasing incidence of head and neck cancer, the healthcare system lacks early diagnosis. The most common causes of carcinoma have been established and studied, including but not limited to tobacco use, alcohol abuse, nutritional deficiency, poor oral hygiene, and viral infection (Deshmukh et al., 2022). These primary and secondary etiologies can both be prevented and treated as patients seek medical attention; however, the focus of interest is the diagnostic approach. The term “diagnostic delay” is the time between the first tangible symptom noticeable to the patient and the actual diagnosis made by a specialist (Scott et al., 2006). This hinders the survival rate of head and neck cancer patients; statistically, a 5-year survival rate is partially accounted by the diagnostic delay, alongside patients’ subjectivity of fear and lack of knowledge about the type of carcinoma itself (Mauceri et al., 2022). Despite the progress in cancer diagnosis and treatment, head and neck cancer is often diagnosed at advanced stages and associated with an approximately 50% mortality rate (Chen et al., 2013). Therefore, there is an urgent need to develop novel approaches that improve and conclude early diagnosis (Masood et al., 2015; Roi et al., 2023).

To date, multiple on-going research studies have tackled the search for early yet accessible diagnostic methods, one of which is using microRNAs (miRNAs) as biomarkers (Nonaka and Wong, 2018). These miRNAs, which are 18–25-RNA-nucleotide long, serving as non-coding regulatory genetic expression, appeared as irregular (either upregulated or downregulated in expression) biomarkers within patients with head and neck cancer (Chen et al., 2013; Ghosh et al., 2020). Growing evidence implicates miRNAs in cancer hallmarks by acting as both tumor suppressors and promoters. Additionally, dysregulated miRNAs have been detected in different body fluids, including blood, saliva, and urine (De Smaele et al., 2010; Yan et al., 2017).

To narrow down the scope of our analysis, oral and pharyngeal, or “oropharyngeal” squamous cell carcinoma (OPSCC), is the focus and inclusive factor. Throughout the meta-analysis process, we compare different liquid biopsy biomarkers, such as blood miRNAs, salivary miRNAs, or a combination of blood and salivary miRNAs, and their roles and likelihood to accurately diagnose the prevalence of OPSCC using statistical software. The purpose of this study is to systematically review published articles within credible and major databases and conduct a meta-analysis to demonstrate the diagnostic role of blood and salivary miRNAs as biomarkers by providing the most comprehensive and updated meta-analysis.

## 2 Materials and methods

### 2.1 Search strategy

This meta-analysis was anticipated to be a randomized systematic review; the methodical research requires strict categorical criteria, which is discussed later, and an unbiased thorough literature search in major databases, such as PubMed, Web of Science, and National Library of Medicine, to include all appropriate and qualified articles regarding different categories of miRNA liquid biomarkers, including but not limited to blood, urine, saliva, and other bodily fluids. More importantly, data collected from these articles must consist of, or be measurable/calculable, sensitivity, specificity, likelihood ratio, true positive (TP), true negative (TN), false positive (FP), false negative (FN), area under the curve (AUC), and changes in miRNA expression. All qualified articles should measure within either oral and/or pharyngeal squamous cell carcinoma to be further included in the selection of abstract and profound data and meta-analysis. Supplementary Appendix S1 provides the terms used for the literature search (Supplementary Table S1).

### 2.2 Eligibility criteria

Only *in vivo* human subjects will be included and considered in the study, which excluded *in vitro* and/or any animal subjects. Some inclusion criteria were classified as non-duplicated, within a 10-year timeframe (2013–2023), including sufficient data (sensitivity, specificity, likelihood ratio, TP, TN, FP, FN, and AUC) either reported or provided through necessary calculation, and OPSCC-specific studies between the concerned subject and qualified controls. This concerns the length of the study, multiple miRNAs of interest within the same study, worldwide location of research, and full-text accessibility.

### 2.3 Data extraction and quality assessment

In the effort to sort each study starting from the beginning of the interested timeframe (2013), information extracted from each study was included and organized into two tables: 1) study characteristics, such as author, year of publication, location of study, number of patients and control subjects, respective miRNA liquid biomarker specimens, and detection method, and 2) miRNA profiles, changes in expression, and sufficient data to complete the analysis (sensitivity, specificity, TP, TN, FP, FN, and AUC). For quality assessment, RevMan (v.5.4) Quality Assessment of Diagnostic Accuracy Studies 2 (QUADAS-2) was used to compare studies across different categories, such as patient selection, index test, and reference standard.

### 2.4 Statistical analysis

The meta-analysis was conducted using Stata (BE, v.18) statistical software. A random-effects model was used to calculate the sensitivity, specificity, positive likelihood ratio (PLR), negative likelihood ratio (NLR), and diagnostic odds ratio (DOR) with a pooled 95% confidence interval (CI). The diagnostic accuracy of miRNAs was assessed using forest plots and summary receiver operating characteristics (SROC). Cochran’s Q test and I^2^ statistic were used to assess the presence of statistical heterogeneity between studies; this helped exclude any category that did not qualify. An I^2^ value of 75% was considered high heterogeneity. Ultimately, the diagnostic accuracy of miRNAs was assessed using the funnel plot, SROC, and the area under the SROC curve (AUC). We also separated blood and salivary miRNAs to assess their individual heterogeneity, with I^2^ of approximately 25% considered low, whereas I^2^ of approximately 50% is intermediate. Lastly, Deeks’ funnel plot of publication bias was included to determine the presence of any unforeseeable bias; a *p*-value of less than 0.05 was considered significant.

## 3 Results

### 3.1 Study selection

**FIGURE 1:**
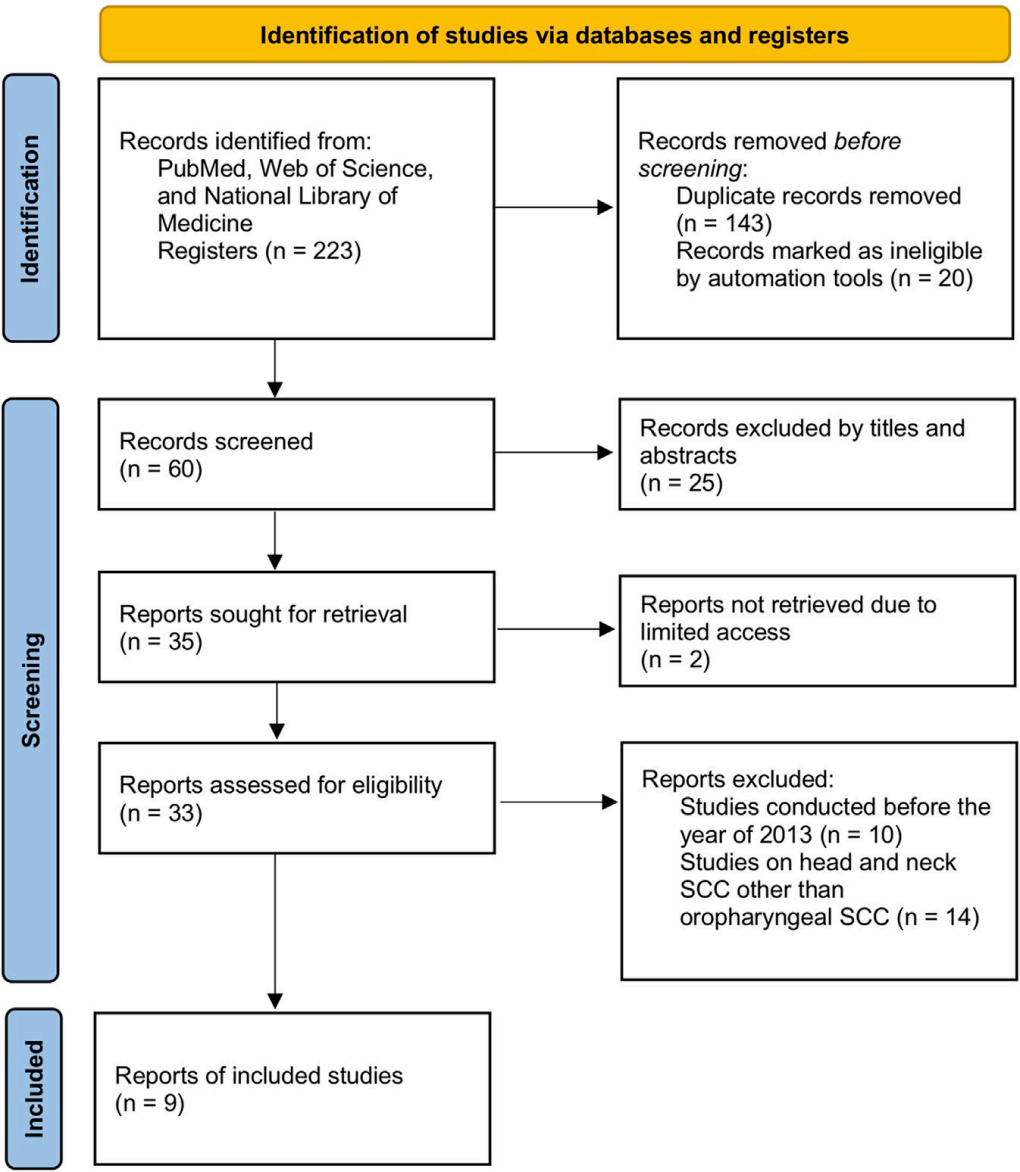
PRISMA flow diagram of the study selection process.

Two investigators retrieved the full text for the qualified articles. No disagreement was observed between the assessors. A total of 143 duplicated articles were removed out of 223 articles initially retrieved from PubMed, Web of Science, and/or National Library of Medicine. After further screening of titles and abstracts, 25 more articles were excluded due to incomplete datasets, and 20 articles were marked as ineligible by automation tools. The remaining 35 articles were thoroughly screened to narrow down to 9 studies, with reasons being a timeframe within 10 years, qualified and specified studies of oral and pharyngeal squamous cell carcinoma, and full-text article assessed for eligibility. The step-by-step flowchart is included to clarify the process (Figure 1). Finally, 9 articles were considered within this meta-analysis consisting of 399 patients with OPSCC with 14 miRNAs of interest.

### 3.2 Study characteristics

Within the included 9 articles, 5 were studies regarding salivary miRNAs, and 4 were studies regarding different types of bodily fluids (ranging from plasma to serum). In total, 14 actual miRNA profiles among those 9 articles were studied within different continents of the world: America (n = 1), Asia (n = 7), and Europe (n = 1). Detailed characteristics, such as the year of publication, location, and number of patients and control subjects, with different specimen origins and detection methods, and more information about each miRNA profile were also extracted and/or calculated for their assessment and are provided in Table 1.

**TABLE 1:** Characteristics of the studies included in the meta-analysis.

		Sample size							
Author, year	Location	Patients	Control	miRNA studied	TP	FP	FN	TN	Sample
Hung et al. (2013)	China	51	12	miR-16	40	1	11	11	Plasma
Momen-Heravi et al. (2014)	United States of America	9	8	miR-27b	8	0	1	8	Saliva
Zahran et al. (2015a)	Saudi Arabia	100	20	miR-21	65	7	35	13	Saliva
Zahran et al. (2015b)	Saudi Arabia	100	20	miR-145	60	6	40	14	Saliva
Zahran et al. (2015c)	Saudi Arabia	100	20	miR-184	80	5	20	15	Saliva
Tachibana et al. (2016)	Japan	31	31	miR-233	21	12	10	19	Serum
Min et al. (2017)	Korea	18	18	miR-146a-5p	14	3	4	15	Saliva
Chang et al. (2018a)	China	114	70	miR-150-5p	69	16	45	54	Plasma
Chang et al. (2018b)	China	114	70	miR-423-5p	67	19	47	51	Plasma
Gai et al. (2018a)	Italy	21	11	miR-412-3p	17	1	4	10	Saliva
Gai et al. (2018b)	Italy	21	11	miR-512-3p	17	3	4	8	Saliva
Shi et al. (2019a)	China	105	60	miR-626	77	18	28	42	Serum
Shi et al. (2019b)	China	105	60	miR-5100	76	17	29	43	Serum
He et al. (2020)	China	45	10	miR-24-3p	29	2	16	8	Saliva

qRT-PCR was used to detect miRNAs for all studies. TP, true positive; FP, false positive; FN, false negative; and TN, true negative.

### 3.3 Quality assessment

**FIGURE 2:**
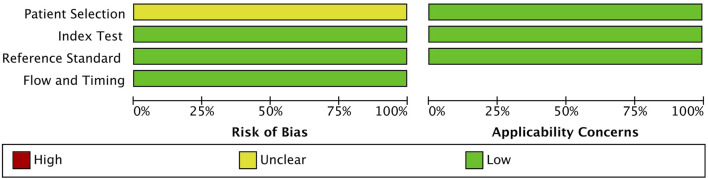
Quality Assessment of Diagnostic Accuracy Studies 2 (QUADAS-2) quality assessment of the 9 included studies.

For quality assessment, QUADAS-2 was used to compare studies across different categories, such as patient selection, index test, reference standard, flow, and timing. The result obtained using RevMan software showed a low risk of bias in the index test, reference standard, flow, and timing; despite an unclear risk of bias in patient selection, the applicability concern was low for that category (Figure 2). Hence, overall, the study is acceptable for the quality assessment.

### 3.4 Meta-analysis

For the initial assessment, both salivary and blood (including both plasma and serum) miRNA biomarkers were combined to determine the sensitivity, specificity, PLR, NLR, DOR, and AUC of all 14 miRNAs. As the number of salivary miRNAs included research was neither balanced nor passed the threshold of heterogeneity, they were unqualified for our meta-analysis; hence, this category was excluded from individualized assessment. Blood-derived miRNAs were qualified, with a high I^2^ value of above 70% for DOR qualification (Liu et al., 2021) considered statistically qualified for heterogeneous characteristics.

**FIGURE 3:**
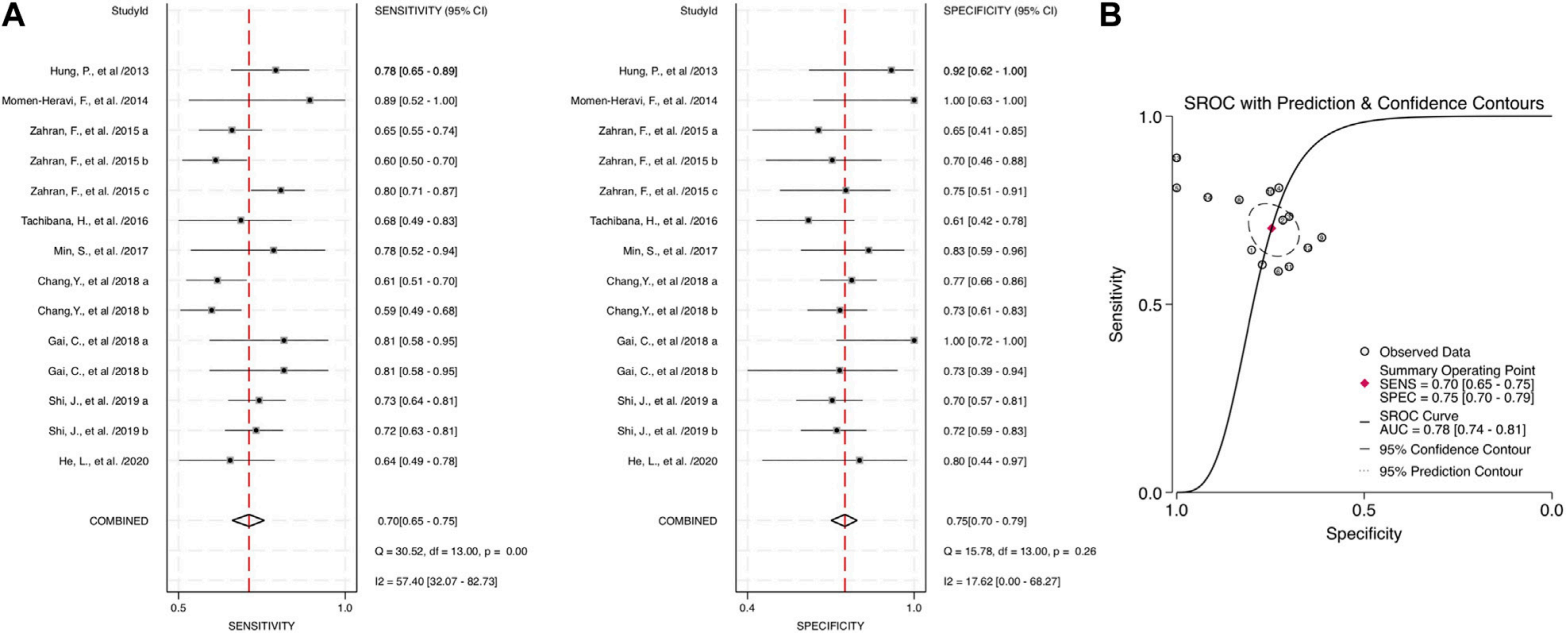
Estimates on diagnostic performances for OPSCC by combined blood and salivary miRNAs. **(A)** Forest plot of the pooled sensitivity and specificity of combined blood- and saliva-derived miRNAs for diagnosing OPSCC. **(B)** SROC curve with estimates of pooled sensitivity and specificity of combined blood- and saliva-derived miRNAs in OPSCC diagnosis. SENS, sensitivity; SPEC, specificity; SROC, summary receiver operating characteristic; AUC, area under the SROC curve.

**FIGURE 4:**
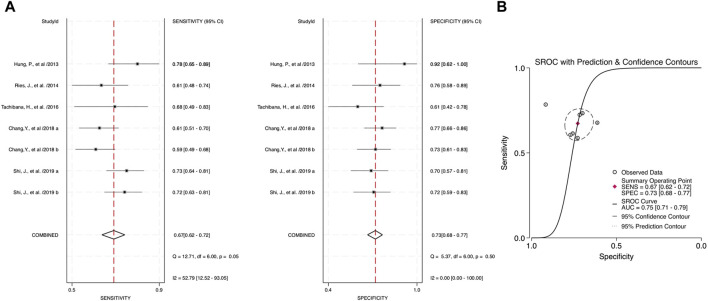
Estimates on diagnostic performances for OPSCC by blood miRNAs. **(A)** Forest plot of pooled sensitivity and specificity for blood-derived miRNAs for diagnosing OPSCC. **(B)** SROC curve with estimates of pooled sensitivity and specificity of blood miRNAs in OPSCC diagnosis.

Through the meta-analysis conducted using Stata software, the forest plot and SROC were generated and used as an illustration for the sensitivity and specificity of each category. Combined salivary and blood miRNAs detected had a sensitivity of 0.70 (95% CI: 0.65–0.75) and a specificity of 0.75 (95%: CI: 0.70–0.79), and the AUC value was 0.78 (95% CI: 0.74–0.81) (Figure 3). For the blood-derived miRNAs, the sensitivity, specificity, and AUC were 0.68 (95% CI: 0.62–0.74), 0.72 (95% CI: 0.67–0.77), and 0.75 (95% CI: 0.71–0.78), respectively (Figure 4).

**FIGURE 5:**
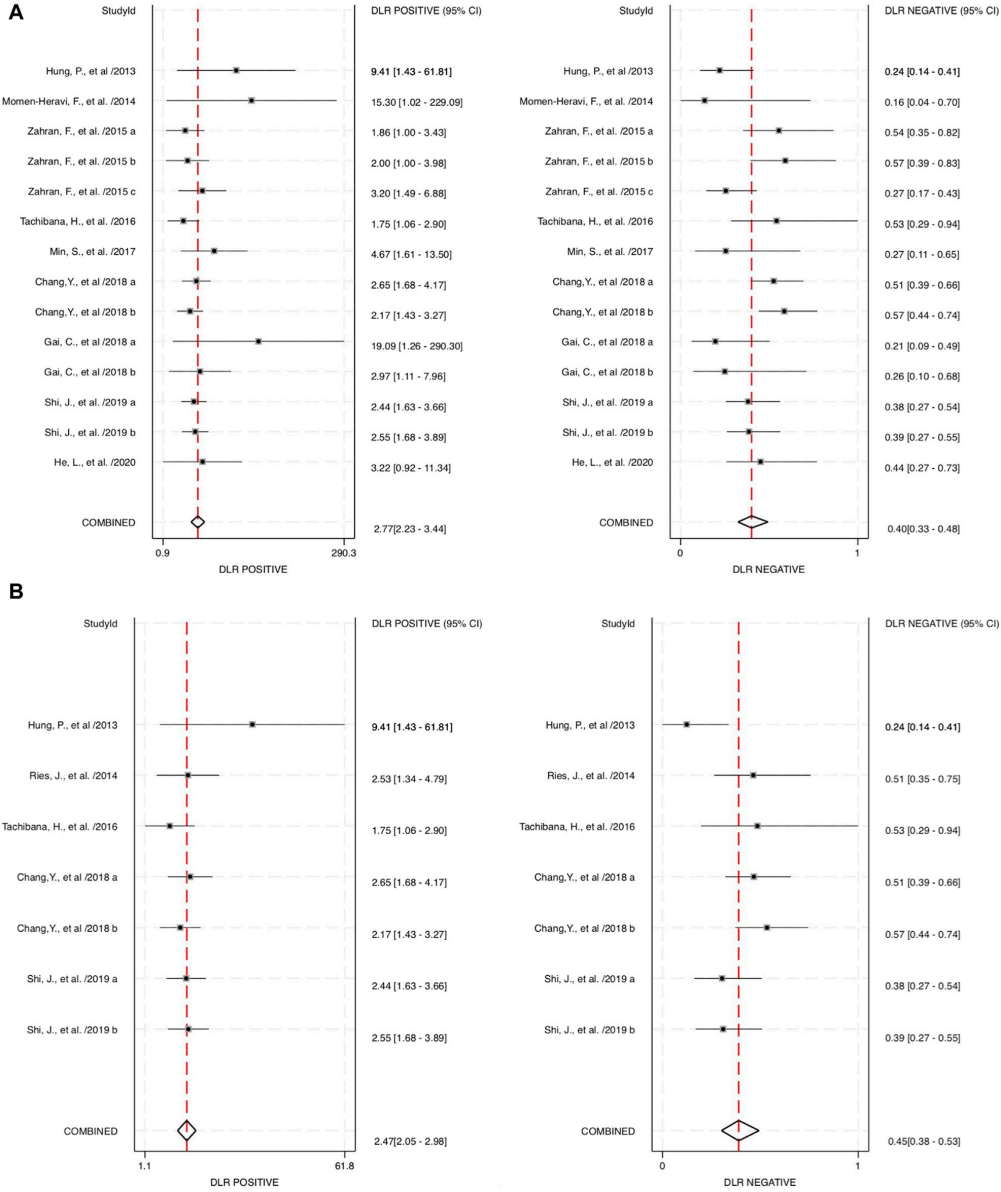
Estimates on diagnostic performances for OPSCC within different categories of miRNAs. **(A)** Forest plot of the diagnostic likelihood ratio for combined blood- and saliva-derived miRNAs for diagnosing OPSCC. **(B)** Forest plot of the diagnostic likelihood ratio for blood miRNAs in OPSCC diagnosis. DLR, diagnostic likelihood ratio.

**FIGURE 6:**
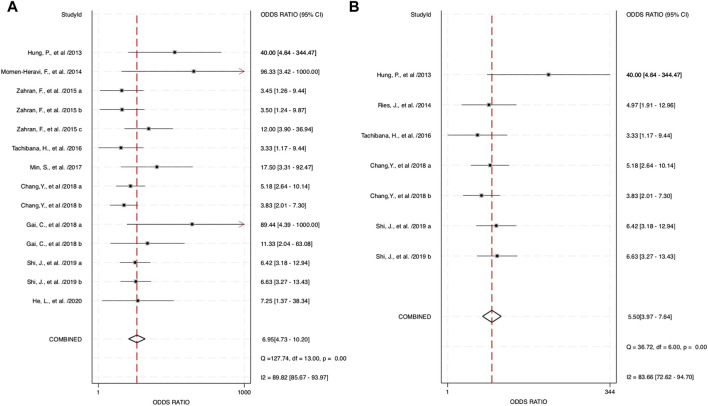
Estimates on diagnostic odds ratio for oropharyngeal squamous cell carcinoma (OPSCC) within different categories of miRNAs. **(A)** Forest plot of the diagnostic odds ratio for combined blood- and saliva-derived miRNAs for diagnosing OPSCC. **(B)** Forest plot of the diagnostic odds ratio for blood miRNAs in OPSCC diagnosis.

The combined blood and salivary miRNAs had a diagnostic PLR and NLR of 2.77 (95% CI: 2.23–3.44) and 0.40 (95% CI: 0.33–0.48), respectively, particularly compared to blood miRNAs that had a diagnostic PLR of 2.47 (95% CI: 2.03–3.01) and diagnostic NLR of 0.44 (95% CI: 0.36–0.53) (Figure 5). Next, the diagnostic odds ratio obtained from the forest plot within Stata software was used as the meta-analyzing tool; the results yielded an odds ratio of 6.95 (95% CI: 4.73–10.20) for combined blood- and saliva-derived miRNAs, and that of blood miRNAs was 5.62 (95% CI: 3.94–8.02) (Figure 6). Together, likelihood ratios and diagnostic ratios showed appropriate and moderate accuracy for each category of diagnostic methods.

### 3.5 Publication bias

**FIGURE 7:**
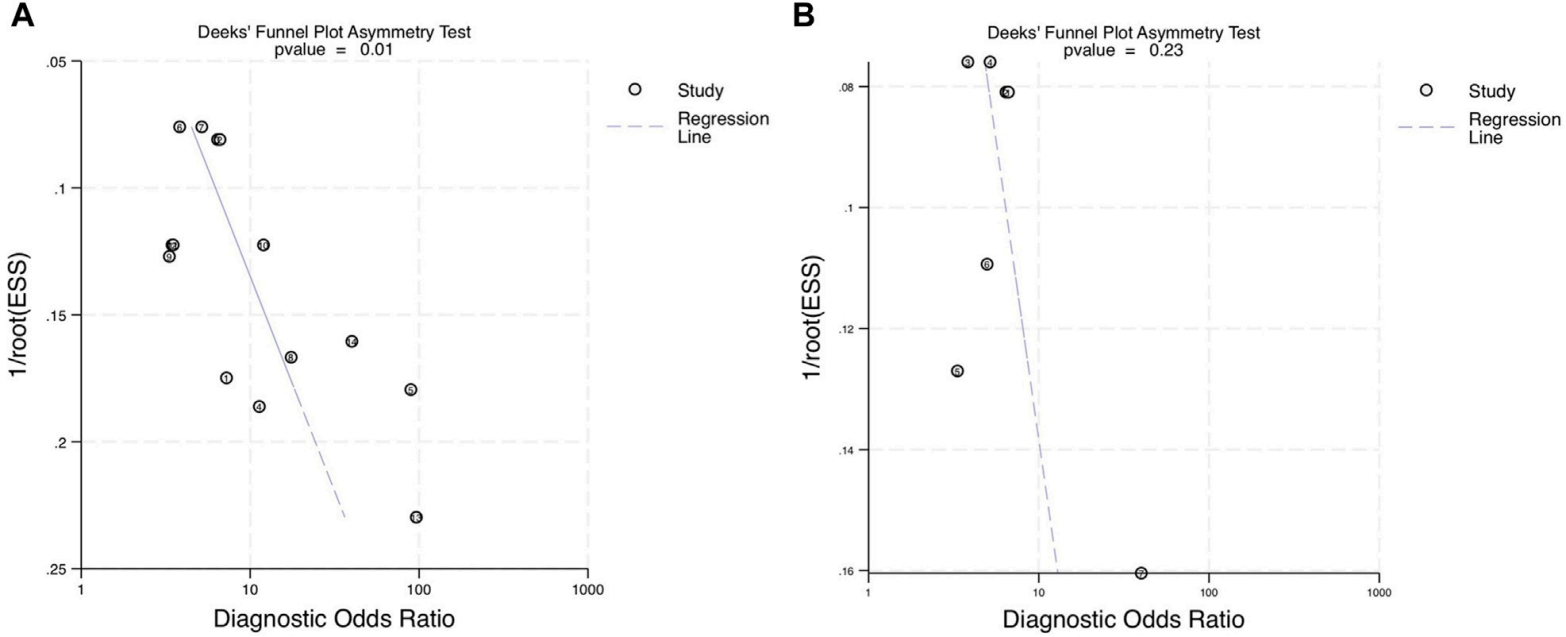
Deeks’ funnel asymmetry plots assessing publication bias within the diagnostic performance of miRNAs for OPSCC. **(A)** Deeks’ plot of the diagnostic odds ratio for combined blood- and saliva-derived miRNAs for diagnosing OPSCC. **(B)** Deeks’ plot of the diagnostic odds ratio for blood miRNAs in OPSCC diagnosis. ESS, effective sample size.

The asymmetry test was used to assess the publication bias in all 9 studies with 14 different miRNAs. Stata generated Deeks’ plots within the established categories. For every included paper, the *p*-value was 0.01 (Figure 7A). The *p*-value showed some significant publication biases within all accounted studies. However, within the 4 blood-derived miRNA studies, the *p*-value was 0.23 (Figure 7B); consequently, the regression line was close to 90°, which indicated no significant publication bias for blood-derived miRNA data.

## 4 Discussion

A previous study stated that the non-invasive method of detection could increase the early diagnosis of oropharyngeal squamous cell carcinoma with the same diagnostic accuracy of a brush biopsy sample (Adami et al., 2017); hence, RNA-derived biomarkers that show dysregulated cell profiles can be the up-and-coming method of detection for cancer. In the realm of this study, the focus is on blood miRNAs or other liquid bodily fluids that can be used as diagnostic measurements for OPSCC detection. We propose that blood- and/or saliva-derived miRNAs can show dysregulation of cell profiles in concurrent patients with oral or pharyngeal squamous cell carcinoma.

The low survival rate of OPSCC patients continues to be an issue despite advanced treatment modalities (Mauceri et al., 2022). A concurrent study inferred the lengthy process of diagnosing and multiple doctor visits until the patient would meet with the specialist to finalize their diagnosis (Liu et al., 2021). Hence, miRNA profiling could be the next novel method of efficient and conclusive diagnosis (Ghafouri-Fard et al., 2020). There should also be a benefit of non-invasive diagnostic assessment, decreasing the going-to-the-doctor fear, which roots the cause of patients failing to initiate the healthcare visit. Therefore, our study can be a significant stepping stone for future studies, using the combination of blood- and salivary-derived miRNAs, which can easily be collected from patients non-invasively to detect early dysregulated miRNA profiles that could potentially cause OPSCC. Ultimately, the database of dysregulated miRNAs that link to OPSCC can be combined and sorted out for future assessment for quick and early detection.

Combined miRNAs of salivary and blood detectable had a sensitivity and specificity of 0.70 and 0.75, respectively. These measurements had moderate-to-high accuracy among the included studies, and the AUC value was 0.78, indicating high accuracy of a quantitative diagnostic test, compared to the single diagnostic measurement of blood-derived miRNAs with a sensitivity, specificity, and AUC of 0.68, 0.72, and 0.75, respectively. These assessment measurements also show moderate-to-high accuracy. Interpreting the value of likelihood ratios, the PLR of both combined and blood miRNAs shows values of more than 2 (2.77 and 2.47, respectively), indicating that a positive outcome (i.e., accurately diagnosed patients) would more than likely happen in the subjects of interest, whereas the NLR would interpretably show a lower chance of misdiagnosing patients in the study, with the values of 0.40 for combined blood- and saliva-derived miRNAs and 0.44 for that of blood. This measurement of test performance concluded a high prediction of a positive outcome for our study, with combined blood and salivary miRNAs having a DOR of 6.95 and blood miRNAs having a DOR of 5.62. These statistical measurements show approximately equivalent results and likelihood among the two concerned categories; hence, more differentiative data are needed to conclude which category could be more preferential.

The significance of the study showed high potential for miRNAs as a novel diagnostic method to detect and accurately confirm the prevalence of oropharyngeal squamous cell carcinoma. This could play a major role in the early detection and appropriate diagnosis of OPSCC in patient in need. Primarily, miRNA profiles of interest were all upregulated as the carcinoma surfaced; this could be one of the limitations of the study as it would narrow down the scope of other downregulated miRNA profiles. Additionally, there are still some limitations that should be taken into consideration, which included but not limited to the following: low subject variation (Momen-Heravi et al., 2014) as less than 20 subjects were taken into consideration in the research; 7 out of 9 studies took place in Asian countries, where a high publication bias in the population incurred (Liu et al., 2021); multifactorial miRNA profiling could affect the outcome of carcinoma expression (Zahran et al., 2015); and lastly, some potential outliers affected the specific *p*-value of combined blood- and salivary-derived miRNAs even though its sensitivity remained significant and relevant. These reasons can yield skewed results in the overall study and lower the quality of the overall diagnostic meta-analysis (Kang et al., 2021). Nevertheless, accumulating evidence reveals the potential of circulating miRNAs as liquid biopsy biomarkers for early diagnoses within the clinical settings.

Our results open more avenues for exploring specific and sensitive miRNAs in populations worldwide to improve the representativeness of patient profiles, especially for the issue of the low survival rate within patients with oral or pharyngeal squamous cell carcinoma. More economical study methods of detection could reduce the cost and increase the accessibility of miRNA screening in a routine healthcare checkup for patients to screen for early stages of OPSCC. Additionally, a large-scale investigation could further choose a specific miRNA profile or a combination of different profiles to improve the specificity and accuracy of OPSCC diagnosis (Mazumder et al., 2019). This further reinforces that a better understanding and databases of such miRNA profiles can be crucial and differentiative in detecting OPSCC early within differential studies during patient routine health checkups.

Combined data from both blood and saliva can be the next novel detection method as salivary miRNAs can be easily retrievable, and “saliva exosomics” is an emerging sub-field of salivaomics research (Nonaka and Wong, 2017). It focuses on the analysis of molecular cargos carried by small (30–100-nm diameter) membrane-enclosed extracellular structures called exosomes. Originating in the endosomal pathway and secreted by almost all cell types, exosomes move through the vasculature to distal sites, including the salivary glands. miRNAs encapsulated in salivary exosomes are stable enough to resist the harsh environment of the human digestive system (Cheng et al., 2019). The lipid bilayer of exosomes acts as a natural barrier to RNases that would otherwise degrade their contents. This makes salivary exosomes an attractive source of material for diagnostic procedures as a non-invasive detecting method.

The key question concerning blood biomarkers is how representative they are of the whole tumor. In this regard, the assessment of biomarkers should be considered in the context of integrity and inclusivity of all tumor features. Given that the tumor cells shed miRNAs directly into saliva, we believe our concurrent analysis approach (i.e., blood plus saliva) will improve the accuracy of liquid biopsy to detect early OPSCC.

Together, our study provides a solid scientific basis for the clinical use of salivary miRNAs. Saliva diagnostics could offer a less invasive and more reproducible alternative (Nonaka and Wong, 2022). Furthermore, routine saliva testing of at-risk patients may reduce cancer-associated mortality (Nonaka and Wong, 2023). Concerted efforts involving collaborations between clinicians, commercial entities, and regulatory bodies are now required to harness the full power of saliva testing in next-generation clinical practice.

## 5 Conclusion

In conclusion, there are higher proofs that combined miRNA profiles of blood and salivary biomarkers were more indicative of diagnostic accuracy for oropharyngeal squamous cell carcinoma than that of blood-derived miRNAs alone. Although more research should be conducted with a more diverse and unbiased selection of subjects, large-scale and widespread testing in subjects could also play a big role in determining the credibility of the study. Ultimately, multifactorial miRNA profiles can also be considered to improve the correlation of different genetic biomarkers playing a role in the presence of OPSCC, and the establishment of a dysregulated miRNA database for future reference of biomarker profiles could be used to help diagnose patients in their early stage.

## Data Availability

The original contributions presented in the study are included in the article/Supplementary Material; further inquiries can be directed to the corresponding author.
