# Supplementary material for: Salivary miRNAs as auxiliary liquid biopsy biomarkers for diagnosis in patients with oropharyngeal squamous cell carcinoma: a systematic review and meta-analysis
Source: Front Genet. 2024 Mar 11;15:1352838. doi: 10.3389/fgene.2024.1352838 (PMC10961377; doi:10.3389/fgene.2024.1352838)
Supplement: Supplementary file 1 [file DataSheet1.docx]

((((((blood[Title/Abstract]) OR (serum[Title/Abstract])) OR (plasma[Title/Abstract])) OR (saliva[Title/Abstract])) OR (body fluids[Title/Abstract])) AND (((((((((("Squamous Cell Carcinoma of Head and Neck"[Mesh]) OR (Squamous Cell Carcinoma of the Head[Title/Abstract] AND Neck[Title/Abstract])) OR (Squamous Cell Carcinoma, Head[Title/Abstract] AND Neck[Title/Abstract])) OR (Carcinoma, Squamous Cell of Head[Title/Abstract] AND Neck[Title/Abstract])) OR (Head[Title/Abstract] AND Neck Squamous Cell Carcinoma[Title/Abstract])) OR (OSCC[Title/Abstract])) OR (oral squamous cell carcinoma[Title/Abstract])) OR (HNSCC[Title/Abstract])) OR (oral cancer[Title/Abstract])) OR (oral tumor))) AND (((((((((((((((((("MicroRNAs"[Mesh]) OR (MicroRNA[Title/Abstract])) OR (miRNAs[Title/Abstract])) OR (Micro RNA[Title/Abstract])) OR (RNA, Micro[Title/Abstract])) OR (miRNA[Title/Abstract])) OR (Primary MicroRNA[Title/Abstract])) OR (MicroRNA, Primary[Title/Abstract])) OR (Primary miRNA[Title/Abstract])) OR (miRNA, Primary[Title/Abstract])) OR (pri-miRNA[Title/Abstract])) OR (pri miRNA[Title/Abstract])) OR (Temporal RNA, Small[Title/Abstract])) OR (stRNA[Title/Abstract])) OR (Small Temporal RNA[Title/Abstract])) OR (pre-miRNA[Title/Abstract])) OR (pre miRNA[Title/Abstract]))) AND (("Sensitivity AND Specificity" OR "False Positive Reactions" OR "False Negative Reactions" OR "ROC Curve" OR "Predictive Value of Tests" OR sensitivity[Title/Abstract] OR specificity[Title/Abstract] OR receiver operating characteristic[Title/Abstract] OR receiver operator characteristic[Title/Abstract] OR likelihood ratio∗[Title/Abstract] OR positive predictive value∗[Title/Abstract] OR negative predictive value∗[Title/Abstract] OR false negative∗[Title/Abstract] OR false positive∗[Title/Abstract] OR true negative∗[Title/Abstract] OR true positive∗[Title/Abstract] OR fn[Title/Abstract] OR fp[Title/Abstract] OR tn[Title/Abstract] OR tp[Title/Abstract]) OR (biomarkers[Title/Abstract]))

Supplemental Table 1. Appendix I. The term used for literature search.
